# A novel thiol-reductase activity of *Arabidopsis* YUC6 confers drought tolerance independently of auxin biosynthesis

**DOI:** 10.1038/ncomms9041

**Published:** 2015-08-28

**Authors:** Joon-Yung Cha, Woe-Yeon Kim, Sun Bin Kang, Jeong Im Kim, Dongwon Baek, In Jung Jung, Mi Ri Kim, Ning Li, Hyun-Jin Kim, Masatoshi Nakajima, Tadao Asami, Jamal S. M. Sabir, Hyeong Cheol Park, Sang Yeol Lee, Hans J. Bohnert, Ray A. Bressan, Jose M. Pardo, Dae-Jin Yun

**Affiliations:** 1 Division of Applied Life Science (BK21Plus), PMBBRC & IALS, Gyeongsang National University, Jinju 660-701, Republic of Korea; 2 Department of Biochemistry, Purdue University, West Lafayette, Indiana 47907, USA; 3 Department of Applied Biological Chemistry, The University of Tokyo, 1-1-1 Yayoi, Bunkyo-ku, Tokyo 113-8657, Japan; 4 Core Research for Evolutional Science and Technology (CREST), Japan Science and Technology Agency (JST), Kawaguchi, Saitama 332-0012, Japan; 5 Department of Biochemistry, King Abdulaziz University, Jeddah 21589, Kingdom of Saudi Arabia; 6 Biotechnology Research Group, Department of Biological Science, Faculty of Science, King Abdulaziz University, Jeddah 21589, Kingdom of Saudi Arabia; 7 Department of Ecological Adaptation, National Institute of Ecology, Seocheon 325-813, Republic of Korea; 8 Department of Plant Biology, University of Illinois at Urbana-Champaign, Urbana, Illinois 61801, USA; 9 Department of Horticulture and Landscape Architecture, Purdue University, West Lafayette, Indiana 47907, USA; 10 Instituto de Recursos Naturales y Agrobiologia, Consejo Superior de Investigaciones Cientificas, Sevilla 41012, Spain

## Abstract

YUCCA (YUC) proteins constitute a family of flavin monooxygenases (FMOs), with an important role in auxin (IAA) biosynthesis. Here we report that Arabidopsis plants overexpressing *YUC6* display enhanced IAA-related phenotypes and exhibit improved drought stress tolerance, low rate of water loss and controlled ROS accumulation under drought and oxidative stresses. Co-overexpression of an IAA-conjugating enzyme reduces IAA levels but drought stress tolerance is unaffected, indicating that the stress-related phenotype is not based on IAA overproduction. YUC6 contains a previously unrecognized FAD- and NADPH-dependent thiol-reductase activity (TR) that overlaps with the FMO domain involved in IAA biosynthesis. Mutation of a conserved cysteine residue (Cys-85) preserves FMO but suppresses TR activity and stress tolerance, whereas mutating the FAD- and NADPH-binding sites, that are common to TR and FMO domains, abolishes all outputs. We provide a paradigm for a single protein playing a dual role, regulating plant development and conveying stress defence responses.

## Abstract (web summary)

YUC6 is a flavin monooxygenase required for the synthesis of the plant hormone auxin. Cha *et al*. discover that in *Arabidopsis*, this enzyme also plays a role in combatting oxidative stress independently of auxin biosynthesis, by acting as a thiol-reductase.

## Graphical abstract

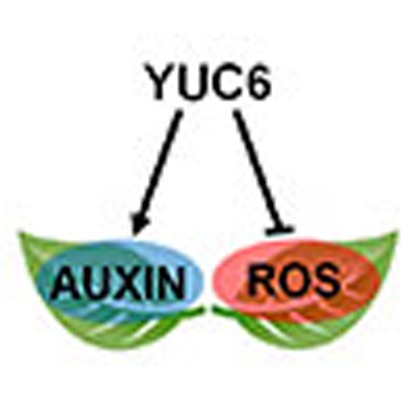

Plant hormones play fundamental roles in regulating growth and development in response to external stimuli and internal cues. The phytohormone auxin regulates many aspects of plant growth and development including apical dominance, tropic responses to light and gravity, root and shoot architecture, vascular differentiation, embryo patterning and shoot elongation^1^. Biosynthesis of IAA, a major auxin in plants, proceeds via tryptophan (Trp)-dependent and Trp-independent pathways whose molecular details are still being elucidated. However, it has become clear that synthesis of IAA from Trp can proceed via four distinct routes that involve indole-3-pyruvic acid (IPA), indole-3-acetaldoxime, indole-3-acetamide and tryptamine (TAM) as pathway intermediates^2^,^3^. It was previously known that TAM, the product of Trp decarboxylation, is a substrate for the YUCCA (YUC) family of plant flavin monooxygenase (FMO) proteins. YUC proteins catalyse the N-hydroxylation of TAM *in vitro* and genetic evidence indicates a role for YUC proteins in IAA biosynthesis^4^,^5^. However, more recent biochemical and genetic experiments have revealed that YUC proteins catalyse also the conversion of IPA, which is produced from Trp by the action of Trp aminotransferase (TAA1/TAR1/TAR2), to IAA *in vivo*^6^,^7^,^8^,^9^.

There are 11 genes encoding YUC proteins in *Arabidopsis thaliana*. Owing to functional redundancy between family members, mutational inactivation of single *YUC* genes does not produce a recognizable phenotype. However, a quadruple *yuc1 yuc4 yuc10 yuc11* mutant displayed severe defects in the formation of floral organs and vascular patterning^10^,^11^. Activation-tagged mutants of several *YUC* genes (*YUC1/4/5/6/7*) and transgenic lines overexpressing these *YUC* genes are characterized by elevated levels of IAA^4^,^5^,^12^,^13^. Consequently, they exhibit high-auxin phenotypes such as elongated hypocotyls, epinastic cotyledons, curled-down leaves, strong apical dominance and delayed dark-induced leaf senescence^4^,^5^,^12^,^13^,^14^,^15^,^16^. Activation-tagged *yuc7-1D* mutant plants are tolerant to drought stress and heterologous overexpression of *YUC6* in potato also improved drought stress tolerance^13^,^17^. *YUC* gene families exist in many plant species, and evolutionarily conserved YUC proteins arguably will have similar functions across species^14^,^17^,^18^. A YUC family protein of rice, *CONSTITUTIVELY WILTED1* (*OsCOW1*), was found to be involved in water homeostasis^19^. The *cow1* mutant has lower root-to-shoot ratio, a developmental defect that probably results in chronic water deficiency and a constitutively wilted phenotype. Developmental defects of the *sparse inflorescence 1* mutant of maize suggest that this YUC protein affects auxin equilibrium. Since IAA pools are maintained at appropriate levels by the coordination of *de novo* biosynthesis, conjugation, degradation and transport^20^, the above results collectively indicate that YUC proteins (*i*) play an important role in auxin balance in several plant species and (*ii*) are directly or indirectly involved in drought tolerance.

Changes in growth rate, development, metabolism and increased antioxidant activities promote adaptation to stress conditions and delay cell death. Reactive oxygen species (ROS) and phytohormones interact and mediate adaptive stress responses. For example, ROS are intermediaries in the pathway leading from abscisic acid (ABA) and salicylic acid (SA) to stomatal closure^21^,^22^,^23^. ROS are also involved in modulating plant growth responses via the gibberellin (GA) pathway^24^,^25^. *GA-stimulated transcript 1* (*GAST1*)-like gene 4 (*GASA4*) is a GA-induced gene that plays a role in promoting GA responses, represses ROS accumulation and has redox activity *in vitro*^26^. Mutations that abolish redox function also cancelled GA responses, indicating that ROS regulate GA signalling. ROS and auxin play major roles in modulating stress-induced developmental changes^27^,^28^,^29^. Exogenous auxin treatments generate ROS transients^30^,^31^ and increase expression of *catalase* (*CAT*) in maize root cells^32^. Arabidopsis mutants with impaired NADPH-dependent thioredoxin reductase (NTR) and glutathione (GSH/glutaredoxin) systems have shown that these antioxidants play key roles in the maintenance of cellular redox homeostasis. For example, the *ntra ntrb cab2* triple mutant of Arabidopsis shows morphological aberrations such as loss of apical dominance, vasculature defects and reduced secondary root production as well as reduced auxin transport, indicating crosstalk between auxin and ROS pathways^33^,^34^. ROS have a role in regulating lifespan and senescence just like auxin, again indicating overlapping functions between ROS and auxin^16^,^35^,^36^.

We report here that Arabidopsis plants overexpressing *YUC6* to accumulate low amounts of ROS exhibit improved antioxidant activity as well as drought tolerance. The phenotype is based on a newly identified FAD- and NADPH-dependent sulfide oxidoreductase activity of YUC6. This novel thiol-reductase (TR) activity is dissociated from the canonical FMO activity by site-directed mutagenesis of a cysteine residue that is most conserved in all Arabidopsis YUC proteins. The observed drought tolerance conferred by YUC6 overexpression is not due to elevated auxin amounts but requires the TR activity of YUC6.

## Results

### *YUC6* overexpression leads to drought tolerance

Plant hormones are known to play important roles in adaptation to environmental stresses, including salinity, temperature fluctuations, water deficit and pathogen infection, due to their effects on growth, development and resource husbandry^37^. Changes in hormone levels or the ability of plants to recognize hormones such as ABA, jasmonic acid, SA, brassinosteroids, cytokinins, ethylene and auxin have all been reported to affect drought tolerance^38^. *YUC6* overexpression increased IAA levels and drought tolerance in transgenic potato plants^18^. To investigate whether the drought tolerance phenotype conferred by *YUC6* overexpression could be attributed to high-auxin content, we first performed experiments to corroborate that overexpression of *YUC6* also increases drought tolerance in Arabidopsis (Supplementary Fig. 1). In these experiments, 3-week-old soil-grown plants were subjected to water withdrawal for 10–14 days, and wilting symptoms in leaves were recorded periodically. Wilting symptoms were significantly greater in leaves of wild-type plants compared with leaves of previously reported *YUC6* overexpression lines such as the *yuc6-1D* and *yuc6-2D* activation mutants and *35S:YUC6* transgenic plants^5^, whereas the loss-of-function *YUC6* mutant, *yuc6-3k*, was more sensitive to water deficiency than the wild-type.

**Figure 1:**
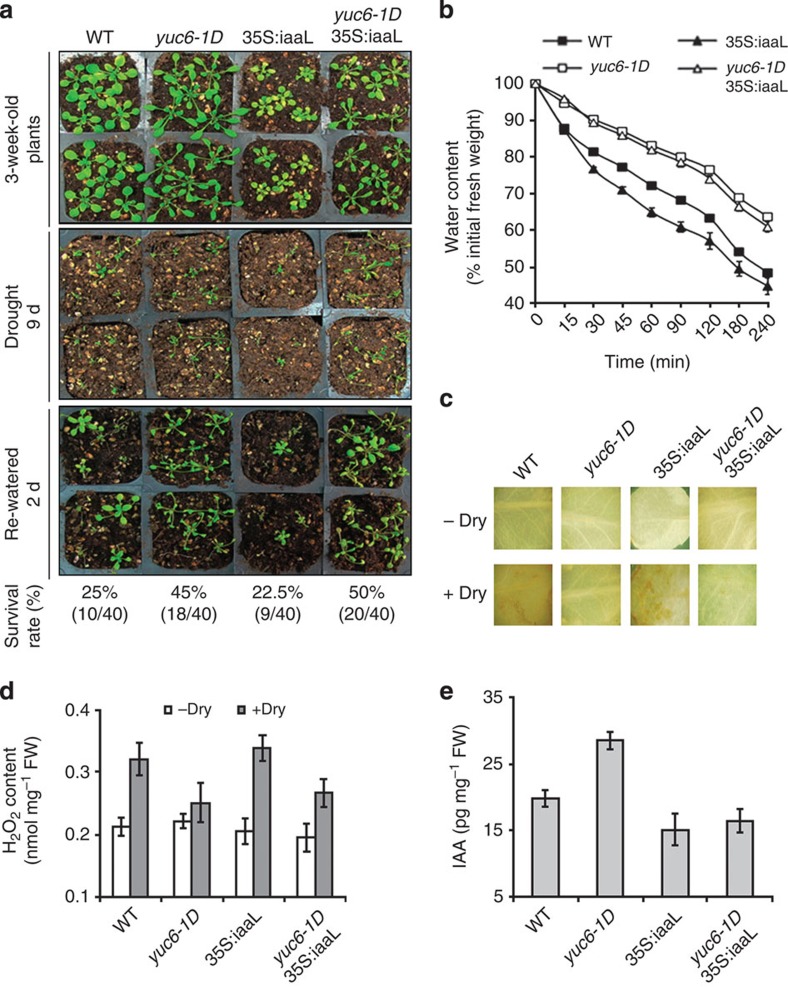
*yuc6-1D* plants exhibit drought stress tolerance that correlates with reduced ROS accumulation. (**a**) Survival assay. The 3-week-old well-watered wild-type (WT, Col-gl), *yuc6-1D*, *35S:iaaL* and *yuc6-1D*X*35S:iaaL* plants (top panel) were grown for 9 days under the same conditions but without irrigation, and then photographed (second panel). The drought-stressed plants were then irrigated. At 2 days after resuming irrigation, the plants were photographed (third panel) and the survival rate (per cent) in each sample was quantified as shown at bottom of the picture. (**b**) Assay of water loss rate. Aerial parts of 3-week-old well-watered soil-grown plants were detached and weighed immediately (0 min). Thereafter, they were allowed to dry naturally under ambient conditions and weighed at the indicated time points. Water content was calculated as the percentage of the fresh weight at time zero. Data represent means±s.e. (*n*=4). (**c**) Visualization of H_2_O_2_ production in response to drought stress. Three-week-old well-watered plants were grown for another 6 day (d) with (−Dry) or without (+Dry) irrigation. Shown are DAB-stained third or fourth rosette leaves of these plants. H_2_O_2_ production is visualized as a dark brown colour. (**d**) H_2_O_2_ accumulations. Aerial parts of 3-week-old plants were detached and air-dried for 4 h (+Dry) or immediately harvested without being air-dried (−Dry). Data represent means±s.e. (*n*=3). (**e**) IAA quantifications. Aerial parts of 3.5-week-old plants were used for quantification of IAA as described in Methods. Data represent means±s.e. (*n*=3).

To investigate the connection between drought tolerance and auxin content, we subjected 3-week-old well-watered wild-type, *yuc6-1D*, *35S:iaaL* and *yuc6-1D*X*35S:iaaL* plants to drought by withholding water for 9 days and then monitored the rate of survival 2 days after re-watering (Fig. 1a). The *yuc6-1D* line overproduces auxin^5^; the *35S:iaaL* line constitutively overexpresses *Pseudomonas savastanoi IAA-lysine synthase* (*iaaL*) from the *CaMV35S* promoter and has phenotypes consistent with a reduction of free IAA level^39^; and the *yuc6-1D*X*35S:iaaL* line has been shown to have almost all the high-auxin phenotypes of *yuc6-1D* restored to wild-type^16^. Direct measurement of IAA content in these genotypes confirmed that *yuc6-1D* plants overproduced auxin compared with WT and *35S:iaaL*, and that coexpression of *35S:iaaL* cancelled auxin accumulation (Fig. 1e). That the *yuc6-1D*X*35S:iaaL* line retained morphological features resembling high-auxin effects, such as elongated petioles (Fig. 1a), suggested that developmentally regulated IAA maxima could still remain locally high and produce outputs reminiscent of high auxin, even though the overall IAA levels were similar to wild-type plants. The survival rate to drought stress of wild-type (25%) and *35S:iaaL* plants (22.5%) was comparable but significantly lower than the survival rate of the *YUC6* overexpression lines, *yuc6-1D* (45%) and *yuc6-1D*X*35S:iaaL* (50%; Fig. 1a). The ability of these plants to tolerate water deficiency was then compared by evaluating water loss from detached aerial plant parts (Fig. 1b). The *yuc6-1D* and *yuc6-1D*X*35S:iaaL* rosettes lost water more slowly compared with wild-type and *35S:iaaL* rosettes. The rate of water loss from detached *yuc6-1D* and *yuc6-1D*X*35S:iaaL* rosettes was similar. These data suggest that drought tolerance phenotype achieved by *YUC6* overexpression is not an inherent function of the coincidental auxin overproduction. Specific temporal and/or spatial patterns of accumulation of IAA may still occur in the *yuc6-1D*X*35S:iaaL* transgenics, in spite of the steady-state levels of IAA in these plants being even lower than that in wild type. However, it appears unlikely that the significant drought resistance observed is associated with the residual high-auxin phenotypes.

ROS, such as free radicals and hydrogen peroxide (H_2_O_2_), are known to accumulate under water-deficit conditions^40^, and we have reported that levels of ROS were lower in leaves of Arabidopsis *YUC6* overexpression plants (*yuc6-1D*) compared with leaves of wild-type plants^18^. Thus, we considered the possibility that drought tolerance of the *yuc6-1D* mutant could be mediated by altered ROS homeostasis. Leaves of soil-grown wild-type, *yuc6-1D*, *35S:iaaL* and *yuc6-1D*X*35S:iaaL* plants that had or had not been subjected to drought stress for 7 days were subjected to histochemical staining using 3,3′-diaminobenzidine (DAB) to detect H_2_O_2_ accumulation. There was no significant increase in ROS production in *yuc6-1D* and *yuc6-1D*X*35S:iaaL* leaves on drought stress, whereas a significant increase in ROS production was observed in wild-type and *35S:iaaL* leaves (Fig. 1c). Quantitation of short-term H_2_O_2_ production in these plants after exposure to air-drying for 4 h showed that H_2_O_2_ contents in *yuc6-1D* and *yuc6-1D*X*35S:iaaL* plants were significantly lower than in wild-type and *35S:iaaL* plants after the dehydration treatment (Fig. 1d). Collectively, these data indicate that drought tolerance of the *yuc6-1D* mutant correlated with the ability to limit ROS accumulation in response to drought stress but not with high-auxin content (Fig. 1a–e).

### The *yuc6-1D* mutant is tolerant to oxidative stress

**Figure 2:**
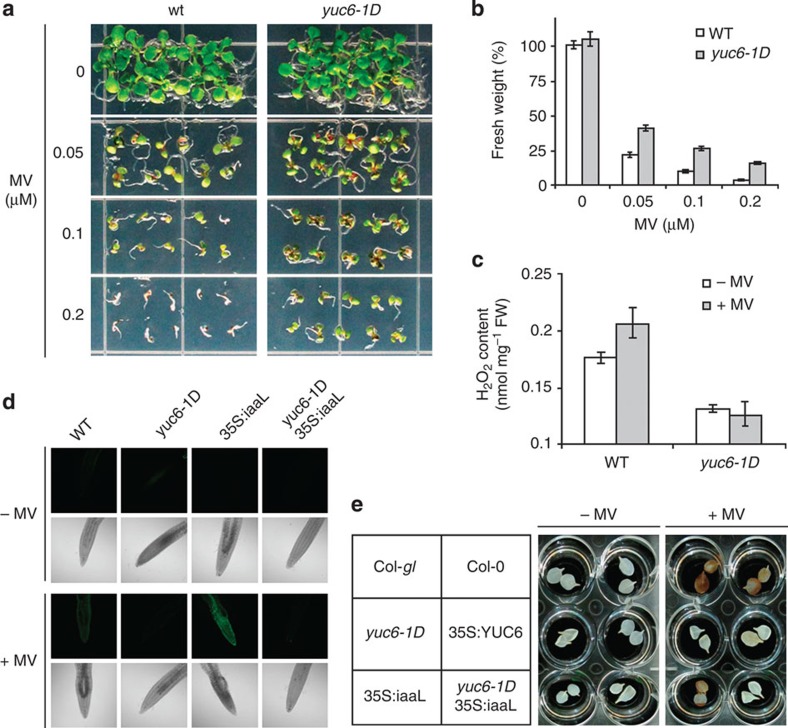
*yuc6-1D* mutant has increased tolerance and decreased ROS accumulation to MV-induced oxidative stress. (**a**) Shown are WT (Col-gl) and *yuc6-1D* plants that were grown on Murashige and Skoog (MS) agar plates containing indicated concentrations of MV for 2 weeks. (**b**) Fresh weight (means±s.e.) of these plants is shown (*n*=10). (**c**) Two-week-old plants grown on MS agar were untreated (−MV) or exposed to 10 μM MV for 4 h (+MV). Bars indicate H_2_O_2_ contents. Data represent means±s.e., *n*=3. (**d**) Fluorescence staining for H_2_O_2_ production. Five-day-old seedlings were untreated (−MV) or exposed to 10 μM +MV for 4 h and stained with H_2_DCF-DA. The fluorescence and white light images of stained root tips are shown. (**e**) Histochemical staining for H_2_O_2_ production. Detached leaves of 2-week-old WT, *yuc6-1D*, *35S:YUC6*, *35S:iaaL* and *yuc6-1D*X*35S:iaaL* plants^5^,^16^ were untreated or treated with 10 μM MV for 4 h. H_2_O_2_ accumulation was visualized by DAB as a dark brown stain.

To test whether reduced level of ROS in *YUC6*-overexpressing plants under drought conditions is specific to water stress, we examined the responses of *YUC6*-overexpressing plants to primary oxidative stress. Methyl viologen (MV) is known to catalyse the production of ROS and promote oxidative stress in plants. Growth of wild-type plants was inhibited more than that of *yuc6-1D* plants on MV-containing media (Fig. 2a,b). Tissue H_2_O_2_ contents were lower in *yuc6-1D* plants compared with wild type even in the absence of oxidative stress (Fig. 2c), which is consistent with previous results^18^. MV treatment increased H_2_O_2_ levels in wild-type but not in *yuc6-1D* plants (Fig. 2c).

To determine whether the inhibition of ROS production by YUC6 was related to auxin metabolism, MV-induced ROS accumulation was determined in roots and leaves by histochemical staining with 2′,7′-dichlorodihydrofluorescein diacetate (H_2_DCF-DA) and DAB, respectively (Fig. 2d,e). ROS accumulation was readily detected in wild-type and *35S:iaaL* plants. However, ROS accumulation was not induced by MV treatment either in *35S:YUC6* leaves or in leaves and roots of *yuc6-1D* and *yuc6-1D*X*35S:iaaL* plants, confirming that *YUC6* overexpression, but not the accompanying elevated auxin content, leads to lower ROS accumulation under oxidative stress. Furthermore, we measured H_2_O_2_ levels after exposure to MV in the loss-of-function *yuc6-3k* mutant, and in the auxin-deficient mutants *taa1 tar2* and quadruple knockout *yuc1,2,4,6*. As shown in Supplementary Fig. 2, the increase in H_2_O_2_ levels in *yuc1,2,4,6* and *yuc6-3k* plants was much greater than in wild-type and *taa1 tar2* plants, suggesting that an unknown property of YUC proteins, separate from auxin biosynthetic activity, might be involved in ROS control under stress conditions.

### YUC6 has a novel TR activity

YUC proteins contain an FMO domain involved in auxin biosynthesis^15^. There are 29 distinct Arabidopsis genes that encode proteins containing motifs typically found in FMOs, of which 11 genes belong to the *YUC* family^15^. YUC proteins share with other FMOs, counting from the N terminus, a FAD (GxGxxG)-binding- site, a FMO-identifying sequence motif (FxGxxxHxxxY/F), a NADPH (GxGxxG/A)-binding site and another sequence (F/LATGY) conserved in FMOs thought to carry out N-oxidation^15^.

**Figure 3:**
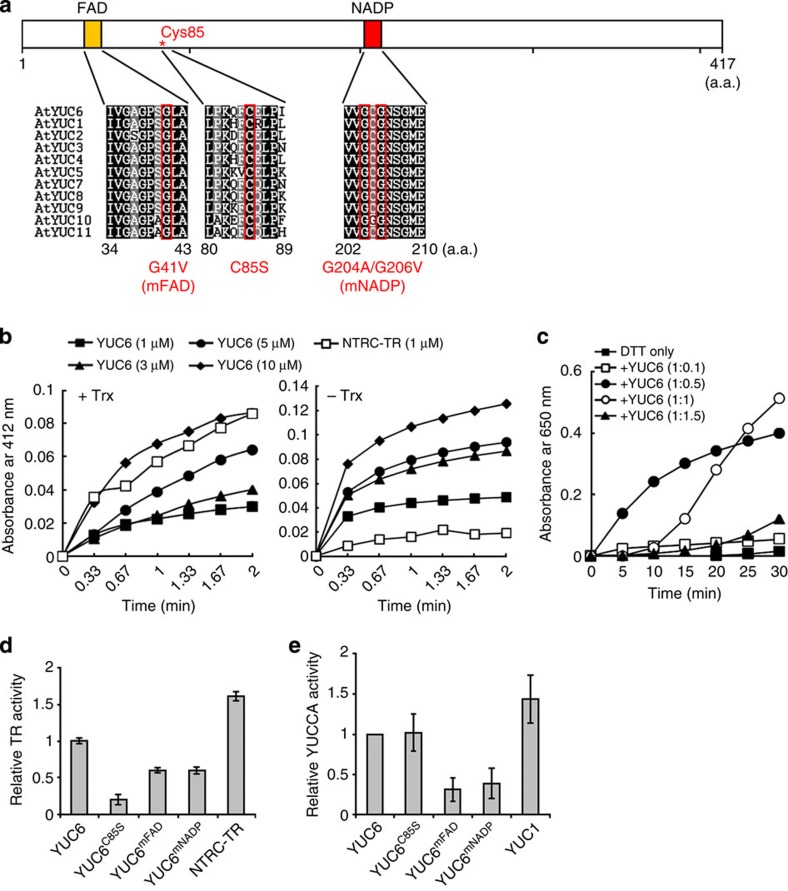
YUC6 possesses TR activity and Cys-85 is essential for its activity. (**a**) Schematic representation of the conserved cysteine and cofactor-binding sites of YUC6. The relative locations of a conserved FAD-binding domain (a.a. 34–43; gold hatch), Cys-85 (red asterisk) and NADPH-binging domain (a.a. 202–210; red hatch) are depicted along the full-length ORF of *YUC6* (417 a.a.; white bar). The FAD- and NADPH-binding sites and the sequence around Cys-85 of all Arabidopsis YUC proteins were aligned, and the residues that were subjected to site-directed mutagenesis are indicated by red boxes. The actual amino-acid substitutions at each location are indicated. (**b**) NADPH-dependent TR activity of purified recombinant YUC6 protein in the presence (+Trx, *left*) or absence (−Trx, *right*) of Trx-h3. TR activity was measured as using DTNB as a substrate and is expressed as increased absorbance at 412 nm. Trx-h3 was added as a reducer in +Trx. Purified recombinant Arabidopsis NTRC TR domain (NTRC-TR) was used as a positive control. (**c**) DTT-dependent insulin reductase activity of recombinant YUC6 protein. Insulin reductase activity is expressed as the increase in absorbance at 650 nm. DTT only refers to a sample containing insulin and DTT but no YUC6. The other samples contained the same amount of insulin with increasing amounts of YUC6 in the indicated molar ratios, in addition to DTT. (**d**) Comparison of the TR activity of WT and mutant YUC6 proteins. TR activity of the purified recombinant YUC6 proteins was measured using DTNB as described above. Relative activity refers the rate of Abs_412nm_ increase for a sample when the rate of Abs_412nm_ increase for WT YUC6 is taken as 1. Recombinant NTRC-TR was used as a positive control for TR activity. Data represent the means±s.e., *n*=3. (**e**) Comparison of the YUC activity of WT and mutant YUC6 proteins. YUC activity using IPA as a substrate was measured using Ehrlich reagent at Abs_585nm_ and relative activity were calculated as same as TR activity. Recombinant YUC1 was used as a positive control for YUC activity. Data represent the means±s.e., *n*=3.

To investigate the possibility that YUC6 carries a direct role in ROS homeostasis, we analysed YUC6 protein sequence by BLAST search to find sequence similarities in addition to the FMO-specific features. YUC6 shares weak sequence similarity (15% identity) to thioredoxin (Trx) reductases (TrxR) from bacteria (for example, *E. coli* TrxB) and plants (Arabidopsis NTRA and barley NTR2; Supplementary Fig. 3). The Trx systems comprise a class of small redox proteins, Trxs, which are kept in the reduced state by specific TrxR in a NADPH-dependent reaction, and they function as antioxidants, in redox regulation of protein function and in cellular signalling^41^. Both of FAD and NADPH are cofactors for FMOs and TrxRs and, therefore, FAD- and NADPH-binding sites are present in YUC6 and TrxR proteins^42^ (Supplementary Fig. 3). Fully conserved Gly residues in the FAD- and NADPH-binding sites are required for cofactor binding and FMO activity^43^. In addition, two conserved Gly residues in the NADPH-binding site of YUC6 (Fig. 3a) have been shown to be essential for the delayed senescence and high-auxin phenotypes of *YUC6* overexpression lines of Arabidopsis^16^.

To follow this lead, we purified bacterially expressed maltose-binding protein (MBP)-tagged YUC6 and assayed for TrxR activity. In the presence of NADPH and Arabidopsis Trx h3, YUC6 (1–10 μM) was able to catalyse the reduction of 5,5-dithio-bis(2-nitrobenzoic acid) (DTNB) in a concentration-dependent manner (Fig. 3b, +Trx). However, YUC6 had about one-tenth the specific activity of the positive control protein, the TrxR domain of NTRC in which the C-terminal Trx domain has been deleted (NTRC-TR)^44^, and YUC6 also displayed TR activity in the absence of Trx h3, whereas NTRC-TR did not (Fig. 3b, − Trx). These results demonstrated TR activity by YUC6 independent of Trx. Recombinant YUC6 also had protein disulfide reductase activity with insulin and dithiothreitol (DTT; 10 mM) as a substrate and a reductant, respectively (Fig. 3c). No increase in turbidity at 650 nm, arising from reduction of insulin and precipitation of the free insulin B chain, was observed in the absence of YUC6. Reduction of insulin was observed at 1:0.5 or 1:1 molar ratios of insulin:recombinant YUC6. Higher proportions of YUC6 were inhibitory, indicating that at high concentrations YUC6 prevents precipitation of free insulin B chains, perhaps by forming soluble YUC6-reduced insulin complexes. This behaviour is a typical property of holdase chaperones. Indeed, an Arabidopsis Trx-like protein that has holdase chaperone activity exhibits the same behaviour as YUC6 in the insulin disulfide reductase assay^45^. Accordingly, recombinant YUC6 protein effectively prevented heat-induced aggregation of malate dehydrogenase (MDH), measured as an increase in turbidity at 340 nm, in a concentration-dependent manner, indicating that it has holdase chaperone activity (Supplementary Fig. 4a). In addition, SDS–PAGE migration analysis revealed that MDH was kept soluble by the addition of YUC6 under thermal denaturing condition at 45 °C, while MDH fully precipitated as aggregates in the absence of YUC6 (Supplementary Fig. 4b). Collectively, these biochemical data indicate that YUC6 functions as a TR.

Most NADPH-dependent TrxRs contain a conserved CxxC motif (Supplementary Fig. 3) that is also found in the catalytic site of many redox-active proteins^42^. YUC6 lacks the characteristic active site CxxC motif of TrxR (Supplementary Fig. 3). However, TRs can perform reactions between their active-site cysteines and cysteines of their disulfide substrates via dithiol or monothiol mechanism^43^. Of the 13 Cys residues present in YUC6, only Cys-85 in the conserved motif C(E/Q)LP (Fig. 3a and Supplementary Fig. 3) is fully conserved among the 11 YUC proteins of Arabidopsis, suggesting that Cys-85 might have an essential role in YUC function. To examine the role of the conserved motifs in both the TR activity domain and the oxidative decarboxylation of IPA catalysed by YUC6 that is related to auxin biosynthesis (representing YUCCA activity), we performed site-directed mutagenesis on the *YUC6* cDNA (Fig. 3a). The FAD-binding site mutant (*YUC6*^*mFAD*^) was generated by a G41V substitution and the NADPH-binding site mutant (*YUC6*^*mNADP*^) was generated by G204A and G206V substitutions. Cys-85 was replaced with Ser to generate the *YUC6*^*C85S*^. The mutant YUC6 proteins were expressed in bacteria as MBP-tagged peptides, purified to homogeneity and assayed for TR and YUC activity *in vitro* (Fig. 3d,e). YUC proteins have been placed downstream of TAA1/TAR converting IPA to IAA^6^,^7^,^8^,^9^, and thus we measured YUC activity using IPA as a substrate. The recombinant proteins YUC6^mFAD^ and YUC6^mNADP^ retained only ∼50% of the TR and YUC activities of the wild-type YUC6 protein, thus confirming that the FAD- and NADPH-binding sites are required for both TR and YUC activities^46^. However, YUC6^C85S^ had almost the same YUC activity as wild-type YUC6 protein but, interestingly, only ∼20–30% of its TR activity. YUC6^C85S^ also had considerably less holdase chaperone activity than wild-type YUC6 protein (Supplementary Fig. 4c). The identification of Cys-85 as essential for the TR- and chaperone-like activities of YUC6 but dispensable for YUC function allowed us to examine the physiological relevance of distinct biochemical activities of YUC6 separately.

### Separate drought tolerance and high-auxin phenotypes

**Figure 4:**
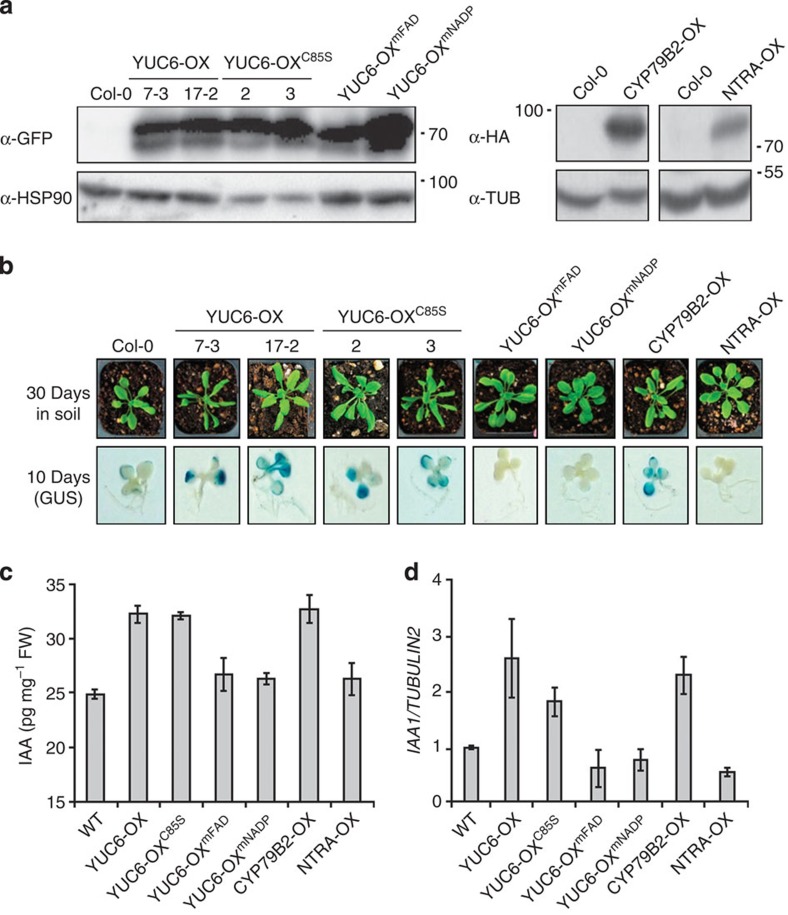
Cys-85 is not required for the high-auxin phenotype of transgenic plants overexpressing *YUC6*. Transgenic lines overexpressing YUC6-YFP (YUC6-OX), YUC6^C85S^-YFP (YUC6-OX^C85S^), YUC6^mFAD^-YFP (YUC6-OX^mFAD^), YUC6^mNADP^-YFP (YUC6-OX^mNADP^), CYP79B2-HA (CYP79B2-OX) and NTRA-HA (NTRA-OX) from the 35S promoter were obtained by transformation of a *DR5:GUS* transgenic line in the Col-0 background (WT). (**a**) Western blots show expression of green fluorescent protein (GFP)- and haemagglutinin (HA)-tagged proteins in the transgenic lines. The tagged proteins were detected with anti-GFP and anti-HA antibodies. α-HSP90 and α-tubulin signals are shown as loading controls. (**b**) One representative 30-day-old transgenic plant of each indicated line is shown (top). GUS staining of 10-day-old seedlings shows elevation of the auxin-responsive *DR5:GUS* activity only in YUC6-OX, YUC6-OX^C85S^ and CYP79B2-OX plants. (**c**) IAA quantifications. Aerial parts of 3.5-week-old plants were used for quantification of IAA as shown in Fig. 1e. Data represent means±s.e. (*n*=3). (**d**) Expression level of *IAA1* and *TUBULIN2* (*TUB*; control) was measured using qRT–PCR in total extracted from 2-week-old plant tissues. Bars represent means±s.e., *n*=3.

The synthetic *DR5* promoter that consists of tandem repeats of an auxin-responsive TGTCTC element has been widely used as a reporter of auxin responses at the cellular level^47^. We transformed a *DR5:GUS* line in the wild-type Col-0 background with constructs for expressing *YUC6, YUC6*^*mFAD*^*, YUC6*^*mNADP*^, *YUC6*^*C85S*^, *NTRA* and *CYTOCHROME P450 FAMILY 79 SUBFAMILY B POLYPEPTIDE 2* (*CYP79B2*) as YFP translational fusion proteins under the control of the *CaMV35S* promoter. *NTRA* was used as a control for high TrxR activity^48^, whereas *CYP79B2*, which encodes a cytochrome P450 that converts Trp to the IAA precursor IAOx, was used as a control for high-auxin biosynthetic activity by a pathway that is genetically distinct from the YUC route^49^. Transgenic lines were screened by western blot analysis, and lines with high expression of the transgene product were selected for further analyses (Fig. 4a). Among these overexpression lines (−OX), YUC6-OX displayed elongated downward curled leaves similar to *yuc6-1D* and other IAA-overproducing plants^4^,^5^, and also had strong GUS staining in leaves compared with untransformed control, indicating high-auxin content (Fig. 4b). YUC6-OX^C85S^ and CYP79B2-OX also displayed elongated downward curled leaves and strong GUS staining in leaves indicating IAA overproduction, as expected, since YUC^C85S^ has about the same level of YUC activity as wild-type YUC6 (Figs 3e and 4b). It was predicted that YUC6-OX^mFAD^ and YUC6-OX^mNADP^ plants, that overexpress catalytically inactive YUC, would have normal auxin levels. Accordingly, leaf morphology and leaf GUS stain intensity in YUC6-OX^mFAD^ and YUC6-OX^mNADP^ was very similar to that of untransformed and NTRA-OX plants. In agreement with the GUS-staining results, auxin contents and transcript level of the IAA-responsive gene *IAA1* were similar in wild-type, YUC6-OX^mFAD^, YUC6-OX^mNADP^ and NTRA-OX plants, whereas they were elevated in YUC6-OX, YUC6-OX^C85S^ and CYP79B2-OX plants, as in *yuc6-1D* (ref. 5; Fig. 4c,d). These results demonstrated that FAD- and NADPH-binding sites of YUC6 were essential for auxin production. These results also showed that the TR activity of YUC6 is not required for auxin production, consistent with results showing that mutation of Cys-85 reduced TR and holdase chaperone activities of recombinant YUC6 without affecting YUC activity (Figs 3d,e and 4b–d and Supplementary Fig. 4).

Next, we examined the importance of the FAD- and NADPH-binding sites and of Cys-85 for ROS scavenging in *YUC6*-overexpressing plants. Untransformed, YUC6-OX, YUC6-OX^mFAD^, YUC6-OX^mNADP^, YUC6-OX^C85S^, CYP79B2-OX and NTRA-OX plants were treated with MV, and ROS accumulation was monitored by H_2_DCF-DA staining in roots and H_2_O_2_ content in aerial parts (Supplementary Fig. 5). A fluorescent signal detecting ROS was observed in roots of untransformed, YUC6-OX^C85S^, YUC6-OX^mFAD^, YUC6-OX^mNADP^ and CYP79B2-OX plants but was barely detectable in YUC6-OX and NTRA-OX plants (Supplementary Fig. 5a). Accordingly, H_2_O_2_ contents in aerial parts of YUC6-OX and NTRA-OX plants increased only marginally on MV treatment, whereas plants expressing YUC6 mutated at Cys-85-, FAD- and NADPH-binding sites, or CYP79B2 showed significant accumulations of H_2_O_2_ (Supplementary Fig. 5b). These results indicate that auxin production was not required for the enhanced ROS-scavenging ability of *YUC6* overexpressors. On the other hand, elevation of cellular TR activity protected against ROS accumulation. Intact FAD- and NADPH-binding sites and Cys-85, which were shown to contribute to TR activity of recombinant YUC6 (Fig. 3d), were also important for ROS-scavenging activity. Importantly, Cys-85 was found to be important for ROS scavenging but not auxin overproduction *in vivo*.

**Figure 5:**
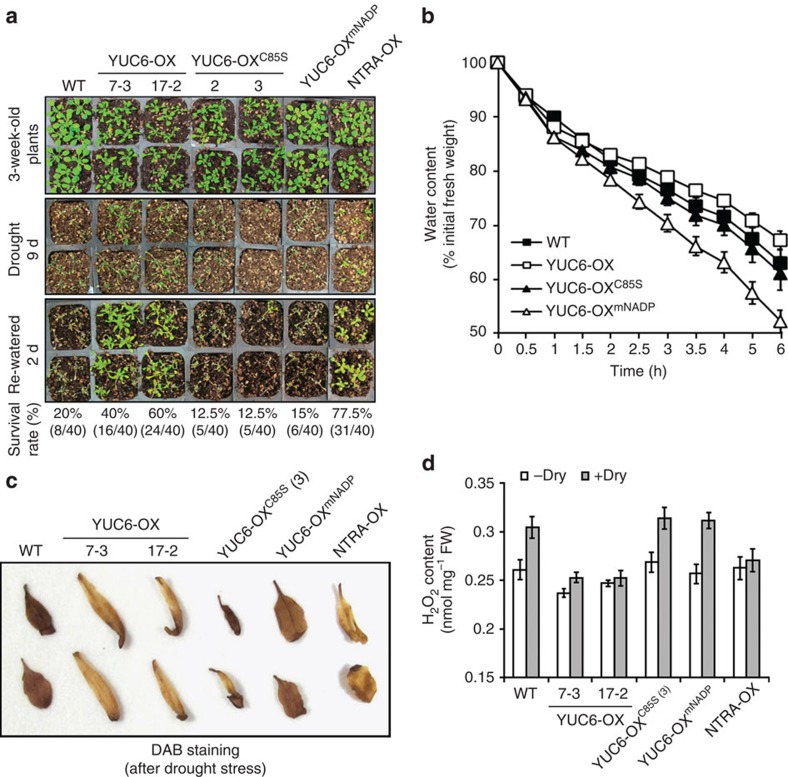
Cys-85 is required for the drought tolerance and low ROS content of *YUC6* overexpression plants. *DR5:GUS* (Col-0) plants (WT) were compared with two independent YUC6-OX transgenic lines (7-3 and 17-2), two independent YUC6-OX^C85S^ transgenic lines (2 and 3), one YUC6-OX^mNADP^ line and one NTRA-OX line in the same background. (**a**) Survival assay was performed exactly as described in Fig. 1a. (**b**) Assay of water loss rate was performed exactly as described in Fig. 1b. Data represent means±s.e., *n*=3. (**c**) H_2_O_2_ production in response to drought stress. Fourth and fifth rosette leaves were harvested after 6-d drought from the plants shown in **a**. The leaves were stained with DAB (1 mg ml^−1^, pH 3.8) for 4 h and H_2_O_2_ was visualized as a dark brown colour. (**d**) Quantification of H_2_O_2_ accumulations. Aerial parts of 3-week-old plants were detached and air-dried for 4 h (+Dry) or immediately harvested without air-drying (−Dry). Data represent means±s.e. (*n*=3).

Next, we investigated the drought tolerance phenotypes of these plants by measuring survival rates of drought-treated plants after a brief re-watering period and by measuring the rate of weight loss in detached leaves that were allowed to air-dry naturally (Fig. 5a,b). We previously reported that NTRA-OX plants exhibit drought tolerance with low accumulation of ROS^50^. Three-week-old well-watered plants were subjected to drought by withholding water for 9 days. Irrigation was resumed and per cent survival was scored 2 days later. YUC6-OX and NTRA-OX had much better survival rates than untransformed control plants (40%, 60% for two independent YUC6-OX lines and 77.5% for the NTRA-OX line, compared with 20% for untransformed). Survival rates of untransformed (WT), YUC6-OX^C85S^ (12.5% for two independent lines) and YUC6-OX^mNADP^ (15%) were lower than that of YUC6-OX plants (Fig. 5a). Accordingly, reduction of water content in detached leaves was slowest in YUC6-OX, marginally faster in untransformed and still faster in YUC6-OX^C85S^ and YUC6-OX^mNADP^ (Fig. 5b). Histochemical staining and H_2_O_2_ contents revealed that tissue ROS content was high in drought stressed leaves of untransformed, YUC6-OX^mNADP^ and YUC6-OX^C85S^ plants, but much weaker in YUC6-OX and NTRA-OX (Fig. 5c,d), indicating that both, the NADPH-binding site and Cys-85, are essential for drought tolerance and low ROS content of YUC6-OX plants.

The cytochrome P450 protein CYP79B2 functions in the auxin-biosynthesis IAOx pathway that is distinct for the YUC-dependent IPA route. Overexpression of *CYP79B2* induced the accumulation of auxin in plants; however, in contrast to YUC6-OX, the ROS content in CYP79B2-OX plants was increased by MV-induced oxidative stress (Fig. 4b–d and Supplementary Fig. 5). Therefore, we further examined whether auxin-overproducing CYP79B2-OX plants showed the output of drought stress tolerance. While YUC6-OX plants displayed drought tolerance, CYP79B2-OX plants were as sensitive as wild-type and YUC6-OX^C85S^ plants (Supplementary Fig. 6a). The water-loss assay supported that CYP79B2-OX shoots were susceptible to dehydration compared with YUC6-OX plants (Supplementary Fig. 6b). This again indicates that auxin overproduction by the overexpression of auxin-biosynthetic pathway genes, such as *YUC6* and *CYP79B2*, does not *per se* lead to drought stress tolerance, which is consistent with ROS accumulation (Figs 4 and 5 and Supplementary Figs 5 and 6).

### Improved ROS scavenging by YUC6 overexpression

**Figure 6:**
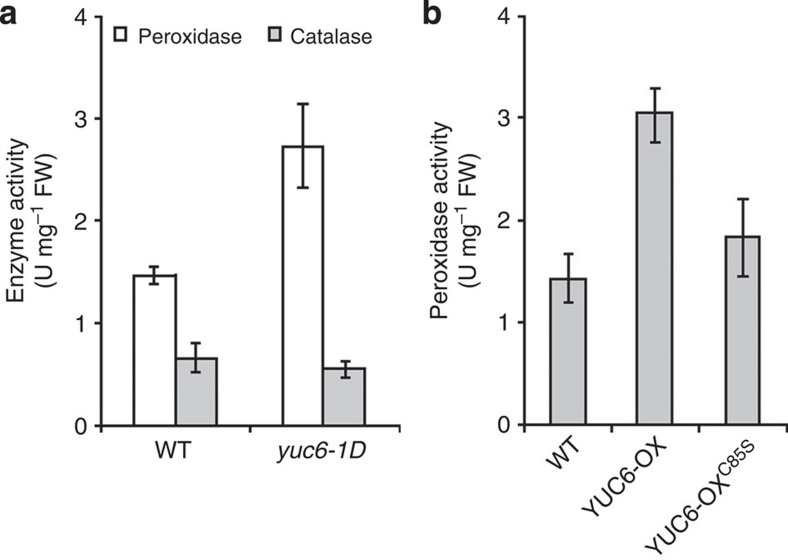
TR activity is required for the increase in tissue peroxidase activity that accompanies *YUC6* overexpression. Two-week-old plants grown on MS agar were used for assay of enzyme activity. (**a**) Bars indicate peroxidase and catalase activities in extracts of *yuc-1D* and isogenic WT plants. Data represent means±s.e., *n*=3. (**b**) Bars indicate peroxidase activity in extracts of isogenic untransformed (WT), YUC6-OX and YUC6-OX^C85S^ plants. Data represent means±s.e., *n*=3.

ROS-scavenging enzymes such as superoxide dismutase, catalase and peroxidase are known to protect against stress-induced ROS accumulation by detoxification of ROS. Microarray analysis revealed enrichment of oxidoreductase and peroxidase transcripts in *yuc6-1D* compared with wild-type plants (Supplementary Table 1). Of these, only At5g59540 is weakly induced, 1.48-fold, by 3-h treatment with 1 μM IAA (ref. 51). In agreement with this observation, peroxidase activity was enhanced in *yuc6-1D* compared with wild-type in absence of stress, whereas catalase activity was similar in both lines (Fig. 6a). By contrast, peroxidase activity in YUC6-OX^C85S^ plants was significantly lower than in YUC6-OX plants (Fig. 6b). These results show that the TR activity of YUC6 mediates the enhanced peroxidase activity that accompanies overexpression of *YUC6*, perhaps by acting upstream of peroxidase expression or activity control. Expression levels of marker genes of the drought response, such as *RD29A* and *DREB1A*, were induced but not significantly different among wild-type, YUC6-OX, YUC6-OX^C85S^ and YUC6OX^mNADP^ plants under drought stress (Supplementary Fig. 7). Together, these results indicate the drought tolerance phenotype associated with *YUC6* overexpression is predominantly mediated by the improved ROS scavenging that is associated with the TR activity of YUC6.

## Discussion

FMO uses NADPH and FAD as cofactors and molecular oxygen as a co-substrate to insert an oxygen atom into an organic compound. In auxin biosynthesis, YUC proteins catalyse the oxidative decarboxylation of IPA to produce IAA. Because reduction of YUC6 by NADPH takes place regardless of the presence of substrates, YUC6 may become an NADPH oxidase using the electrons from NADPH to convert oxygen into hydrogen peroxide. About 4% of the YUC6-catalysed *in vitro* reaction is uncoupled and results in H_2_O_2_ production^9^. Consequently, YUC6 activity has to be tightly regulated. Since YUC proteins conduct oxidative reactions, it is perplexing that YUC6 also demonstrates TR activity and that *YUC6* overexpression prevents rather than enhances ROS production. We and others^9^ have noticed the weak sequence similarities between YUC proteins and flavin-dependent reductases (Supplementary Fig. 3), suggesting that some aspects of the YUC function are similar to reductases. Indeed, our results clearly demonstrate that YUC6 is endowed with NADPH-dependent TR activity and that this function requires Cys-85. The TR activity of YUC6 conveys ROS protection and stress tolerance independently of its activity in auxin biosynthesis.

**Figure 7:**
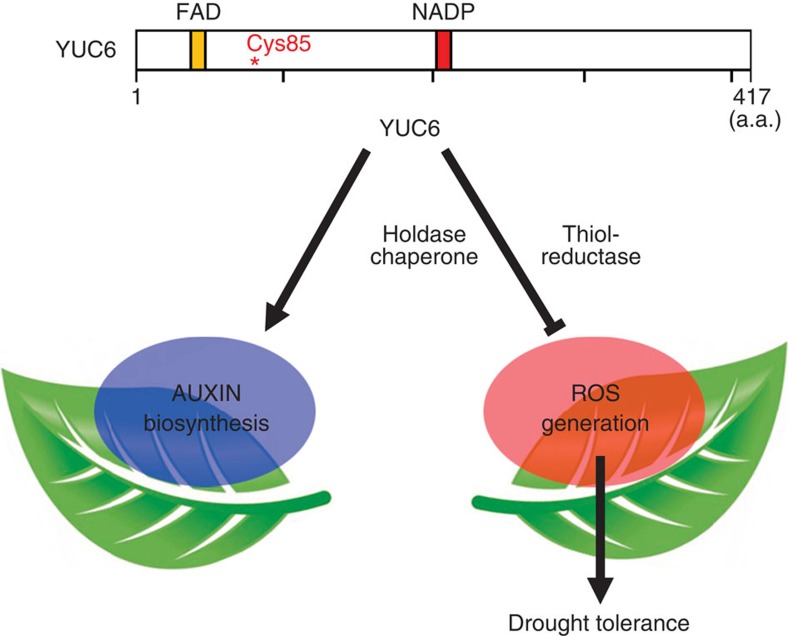
Model describing YUC6 structure–function relationships. YUC6 functions in auxin biosynthetic pathways through its YUC activity. YUC6 also possesses TR activity, thereby reducing ROS content. FAD- and NADPH-binding sites are essential for both YUC and TR activities. Cys-85 is required only for TR activity. In addition, YUC6 displays holdase chaperone activity in a Cys-85-dependent manner. Thus, as an auxin biosynthetic enzyme YUC6 functions in plant development and as a TR-like (and chaperone) protein it functions in ROS regulation and protection against oxidative and drought stresses.

We have incorporated our results into a model (Fig. 7) to explain increased drought tolerance due to overexpression of *YUC6*. Owing to its TR activity, overexpression of *YUC6* reduces ROS content of cells, at least in part by increasing the activity and/or stability of peroxidases and the concerted upregulation of other redox homeostasis genes (Supplementary Table 1). The FAD- and NADPH-binding sites of YUC6 are required for the YUC activity (oxidative decarboxylation of IPA) that leads to auxin biosynthesis. Equally, it is important for the TR activity that improves ROS scavenging and promotes drought tolerance. However, Cys-85 is required only for the TR function of YUC6. The uncoupling of YUC and TR activities in the C85S mutant protein demonstrates that YUC6 confers drought tolerance to plants by a mechanism that is independent of auxin biosynthesis. Thus, YUC6 provides a novel paradigm of a single protein that acquires two related yet distinct functions that share catalytic modules^52^. Each function leads to a separate output.

YUC6 provides for the crosstalk point between adaptive responses to oxidative stress and hormone biosynthesis. Plant hormones are known to play important roles in the adaptation to environmental stresses due to their effects on growth, development and resource husbandry^53^. Although an intricate signalling network is emerging in which ROS, NOS and antioxidants cooperate with hormones to mediate adaptation to environmental cues, evidence for a role of auxins in improving drought stress tolerance has so far been limited. Auxin antagonizes ethylene action by inhibiting H_2_O_2_ accumulation, thus preventing abscission^54^. Exogenous auxin treatment generates ROS transients^30^,^31^ and increases transcription levels of *catalase* (*CAT*) in maize root cells^32^. On the basis of these data, it can be predicted that auxin-overproducing plants will overaccumulate ROS and thus become sensitive to oxidative stress, which is antithetical to our observation that the auxin-overproducing *yuc6-1D* or *35S:YUC6* plants accumulate lower levels of ROS in the absence or presence of oxidative stress and are more tolerant to drought or oxidative stress (Figs 1, 2, 4 and 5 and Supplementary Figs 1, 2, 5 and 6). Furthermore, ROS levels in the quadruple mutant *yuc1,2,4,6* and loss-of-function *yuc6-3k* mutant plants were much greater than in the auxin biosynthesis mutant *taa1 tar2*. The converse situation, where auxin accumulation was driven by the auxin biosynthetic protein CYP79B2 involved in the IAOx pathway that is independent of the YUC-dependent IPA route, also supported the disconnection of IAA levels and stress tolerance. Overexpression of *CYP79B2* produced plants exhibiting overproduction of IAA, while they failed to reduce ROS contents and remained sensitive to drought stress (Fig. 4b–d and Supplementary Figs 5 and 6). Taken together, our data clearly show that the drought tolerance that results from *YUC6* overexpression is not connected to auxin overproduction. Rather, it is associated with the impact of an enhanced TR activity of YUC6 on cellular ROS levels. Since overexpression of either *YUC7* or *YUC6* increases drought tolerance^13^,^18^ and Cys-85 is conserved in all YUC proteins (see below), it is likely that other YUC proteins could have TR activity as well. Indeed, the drought stress phenotype imparted by YUC-family proteins is not restricted to YUC6. The activation-tagged mutant *yuc7-1D* and *35S:YUC7* overexpression lines exhibited phenotypic changes similar to those observed in auxin-overproducing mutants and were resistant to drought^13^. Rice *CONSTITUTIVELY WILTED1* (*OsCOW1*) encodes a protein with homology to YUCs. Inactivation of Os*COW1* results in constitutively wilted leaves and water deficiency^19^. Our data provide a mechanistic explanation for these phenotypes.

We have unveiled a novel TR activity previously unrecognized in YUC proteins, for which Cys-85 is essential. Canonical NADPH-dependent TrxRs (NTRs) of land plants have two neighbouring catalytic cysteines at a conserved redox active site CxxC^43^. However, the CxxC motif is not found and only Cys-85 (using the YUC6 numbering) is fully conserved in all YUC proteins. This might lead to a suggestion that the TR activity of YUC6 proceeds by a different catalytic mechanism. NTRs that lack the CxxC redox motif are also found in prokaryotes; however, mechanistic details of catalysis remain unexplored^43^. Large NTRs and ferredoxin:TrxR have conserved catalytic cysteines forming various motifs that deviate from the CxxC motif^43^. Apart from the essential Cys-85, Cys-63 is also conserved in Arabidopsis YUC proteins, except for YUC1 and YUC10, and fully conserved in all other YUC proteins of higher plants whose sequences are available. Moreover, Cys-63 is embedded in the motif CIASLW that is well conserved in YUC proteins, which is suggestive of functional relevance (Supplementary Fig. 3). Furthermore, Cys-205 is also conserved in all YUC proteins except YUC10; however, it is placed between the essential Gly residues conforming the NADPH-binding site and it is not likely to hold a catalytic function. Conceivably, Cys-63 together with Cys-85 may constitute the redox-active disulfide of YUC6. Structural studies will be needed to elucidate whether these conserved cysteines come in close proximity in the three-dimensional (3D) structure of folded YUC proteins.

Drought stress, alone or in combination with other stresses, leads to ROS accumulation^55^. ROS are signalling intermediates required for normal plant growth and development and also for inducing defensive stress responses. For example, ROS signalling in plant cells is known to regulate stomatal closure and developmental processes that protect against stress conditions^22^,^55^. However, high concentrations of ROS are toxic and induce cell death. ROS accumulation reflects the balance between ROS generation and scavenging. Studies with transgenic plants and comparisons of drought-tolerant and -sensitive cultivars have shown that the control over ROS production and scavenging in chloroplasts, mitochondria, peroxisomes and the apoplast represent important components supporting drought tolerance^55^. Many enzymes and compounds have overlapping functions in ROS scavenging. Enzymes, such as superoxide dismutase, catalase, ascorbate peroxidase, GSH peroxidase and Trx peroxidase (peroxiredoxin, PrxR), are important for ROS detoxification. They have been shown to play important roles in protection against drought stress^55^. TRs, such as the chloroplast-localized NTRC reduces 2-Cys-containing peroxiredoxins that scavenge H_2_O_2_, constituting an essential process for chlorophyll biogenesis, protection of chloroplasts against oxidative damage and protection of plants against drought stress^37^,^56^,^57^. TrxR in other cell compartments secure the constant supply of reduced Trx needed in plant protection against oxidative stress^42^. Thus, the TR activity of YUC6 could function in activating redox systems to scavenge ROS produced under water deficit, as exemplified by the greater peroxidase activity in YUC6-OX and *yuc6-1D* plants (Fig. 6). However, the contribution of YUC6 to ROS control appears to be insufficient to account for the large increase in stress tolerance that plants overexpressing *YUC6* exhibit and presumably other stress protection mechanisms beyond ROS scavenging must be at play, as we argue below.

Chaperones are the key components of many cellular defense systems. Several proteins that are involved in redox homeostasis act as chaperones to prevent unfolding or aggregation via oxidative formation of disulfides in substrate proteins under stress conditions^45^,^58^,^59^. The TR activity of YUC6 may provide a general protection against stress-induced oxidative damage of sensitive proteins. In addition, YUC6 itself exhibits chaperone activity that is dependent on the TR activity (Supplementary Fig. 4). Arabidopsis NTRC displays a heat-mediated holdase chaperone function that is responsible for the increased thermotolerance of *NTRC*-overexpressing plants^60^. Similar to YUC6, NTRC displays holdase activities. Likewise, the holdase function of HSP90 prevented aggregation of the target protein ZEITLUPE, thereby protecting the circadian clock at elevated temperature^61^. Conceivably, YUC6 could also limit oxidative damage owing to its intrinsic holdase-like activity on redox-sensitive protein targets.

Reversible changes in the redox state of structural or catalytic SH groups in target proteins are well suited to control protein function, and thiol-redox control is emerging as a ubiquitous regulatory mechanism in signal transduction. Hence, high hierarchical regulatory roles may be expected for proteins with TR activity. In mammalian systems, several regulatory intermediaries in cellular signalling pathways are regulated by the cellular redox status. For instance, the apoptosis-promoting kinases ASK1 and MPK38 are under redox regulation through Trx binding. Reduced Trx, but not the oxidized form, makes an inhibitory complex with ASK1 and MPK38, in keeping with the general notion of increased ROS as part of the mechanism for induction of apoptosis^42^,^62^. These precedents suggest that there are likely to be a number of yet-to-be-identified proteins critically involved in stress signalling pathways in plants that may be regulated by the cellular redox status. Protein kinases ANP1 of Arabidopsis and NPK1 of tobacco are the closest homologues of ASK1 in plants. Transgenic tobacco plants that express a constitutively active NPK1 display enhanced tolerance to multiple environmental stress conditions without activating drought, cold and ABA signalling pathways; instead, these plants have a combined overproduction of glutathione *S*-transferases and heat-shock proteins^63^. This is reminiscent of our observation that YUC6 overexpression leads to drought tolerance without enhanced expression of marker genes of the drought response, such as *RD29A* and *DREB1A* (Supplementary Fig. 7), while peroxidase activity and ROS homeostasis genes were upregulated in YUC6-OX plants (Fig. 6 and Supplementary Table 1). In Arabidopsis, constitutively active ANP1 mimics the H_2_O_2_ effect in protoplasts and initiates the MAPK cascade that induces stress-responsive genes^63^. Interestingly, NPK1 and ANP1 function in MAPK pathways that repress activities of several promoters responsive to auxin^63^,^64^, presumably to mount osmotic stress tolerance while suppressing auxin-induced cell cycle progression, thereby contributing to stress-linked growth retardation^24^. TrxRs NTRA and NTRB together with GSH-dependent thiol-reduction pathways regulate developmental processes through the modulation of auxin signalling; however, the precise mechanism and proteins involved remain unknown^33^,^34^. These observations provide molecular links between oxidative stress, redox homeostasis and auxin signal transduction. The newly discovered TR activity of YUC6 may be required for the fine-tuning of auxin-dependent growth regulation on stress perception and the promotion of the adaptive response through the control of reduced/oxidized ratios for target proteins.

## Methods

### Plant materials and growth conditions

The *A. thaliana yuc6-1D* line was in the Col-*gl* background, and all other lines were in the Col-0 background^5^,^16^ (Fig. 1 and Supplementary Figs 1 and 2). Isogenic wild-type lines were used for every experiment. Seeds of the sesqui-mutant lines *WEI8-1(TAA1)/wei8-1(taa1) tar2-1/tar2-1* and *yuc1/yuc1 YUC2/yuc2 YUC4/yuc4 yuc6/yuc*6) were kindly provided by ABRC (stock no. CS16414) and Dr Yunde Zhao, respectively. The seeds were sowed and seedlings emerging manifesting the anticipated phenotypes of the double *taa1 tar2* or quadruple *yuc1,2,4,6* mutants (dwarfism and reduced apical dominance) were selected for further experimentation.

To construct transgenic YUC6-OX, YUC6-OX^C85S^, YUC6-OX^mFAD^, YUC6-OX^mNADP^, CYP79B2-OX and NTRA-OX lines, open reading frames (ORFs) of Arabidopsis *YUC6* (At5g25620), the *YUC6* mutants (see ‘Generation of mutated YUCCA6 constructs'), *CYTOCHROME P450 FAMILY 79 SUBFAMILY B POLYPEPTIDE 2* (*CYP79B2*, At4g39950) or *NADPH-DEPENDENT THIOREDOXIN REDUCTASE A* (*NTRA*, At2g17420) were cloned into the donor vector (pDONR-221) and subsequently moved into the pEarleyGate 101 vectors using the recombination-based Gateway cloning system (Invitrogen) according to the manufacturer's instructions. The constructs were introduced into *Agrobacterium tumefaciens* (GV3101) and then into a transgenic *DR5:GUS* line (Col-0 background) via the floral-dip transformation method. Transformants were selected using BASTA, and homozygous lines were verified by western blot analysis using α-haemagglutinin antibody (1:1,000, Roche) and α-green fluorescent protein (1:3,000, Abcam) antibody.

Seeds were surface-sterilized with 30% bleach for 5 min, washed five times with sterile distilled water and incubated for 2 days at 4 °C before sowing. Plants were grown under a 16-h/8-h light–dark cycle at 23 °C in a growth chamber, either on soil or in Petri dishes on Murashige and Skoog medium containing 20 g l^−1^ sucrose and 0.6 % (w/v) agar.

### MV and drought stress treatments

For MV treatments, either 5-day-old seedlings (for H_2_DCF-DA staining), detached leaves of 2-week-old plants (for DAB staining) or 3-week-old (for H_2_O_2_ content) were treated with 10 μM MV by floating on water. For studying the effect of MV on growth, plants were germinated and maintained on MV-containing media.

For drought stress treatments, well-watered soil-grown 3-week-old plants were used. Irrigation was stopped for 9 days and then resumed. Survival rates were calculated by counting the numbers of the green, healthy plants.

For measuring water loss and H_2_O_2_ accumulation, aerial parts of soil-grown 3-week-old plants were detached and allowed to dry at room temperature and humidity. Fresh weight of each plant was measured at indicated time points.

### Amino-acid sequence analyses

The deduced amino-acid sequences of Arabidopsis YUC family proteins and NTRs (Arabidopsis NTRA; barley NTR2; *E. coli* TrxB; green alga ferredoxin-NADP reductase) were retrieved from the GenBank database and aligned using the multiple alignment method of ClustalX and GeneDoc. TR and FMO domains were identified using conserved domain search in NCBI-BLAST (http://blast.ncbi.nlm.nih.gov/).

### Site-directed mutagenesis

The forward and reverse primers for each mutation are listed in Supplementary Table 2. Site-directed mutagenesis of the *YUC6* ORF was performed using Pfu Turbo DNA polymerase (QuickChange Site-Directed Mutagenesis Kit; Stratagene) according to the manufacturer's instructions. pMAL-c2-*YUC6* (ref. 5) was used as the template. Mutations of conserved residues in the TR (Cys-85), FAD-binding site (Gly41) and NADPH-binding site (Gly204/Gly206) of *YUC6* were generated by C85S (*YUC6*^*C85S*^), G41V (*YUC6*^*mFAD*^) and G204A/G206V (*YUC6*^*mNADP*^)^16^ substitutions, respectively. The PCR product was transformed into *E. coli* and fidelity was confirmed in the recovered plasmid using DNA sequencing.

### Expression and purification of recombinant YUC6 proteins

The pMAL-c2-*YUC6* constructs for expression of wild-type and mutant YUC6 as MBP-fusion proteins were transformed into *E. coli* BL21 cells. Protein expression was induced at 30 °C by the addition of 0.5 mM isopropyl-1-thio-β-D-gal-actopyranoside. After 4 h of isopropyl-1-thio-β-D-gal-actopyranoside treatment, cells were harvested. Cells were disrupted by sonication with 1% Triton X-100 and cell debris was removed by centrifugation at 13,500*g* for 10 min. MBP-fusion proteins were purified with affinity chromatography using amylose resins (New England Biolabs).

### DTNB reductase assay

TrxR activity was measured at 23 °C using DTNB as the final disulfide substrate. The reaction mixture contained 150 mM potassium phosphate (pH 8.0), 3 mM EDTA, 5 mM DTNB (Sigma-Aldrich), 200 mM NADPH (Sigma-Aldrich), and in the absence or presence of Arabidopsis thioredoxin h3 (Trx, 1 μM). The reaction was started by the addition of purified recombinant wild-type and mutant YUC6 proteins (as MBP-fusion proteins) and the increase in absorbance at 412 nm was monitored using a ultraviolet–visible spectrophotometer (Beckman Coulter). One unit of TR activity of wild-type YUC6 was defined as Δ412 nm at the last time point (2 min), and relative activity of mutant YUC6 proteins was calculated compared with that of wild-type YUC6 protein.

### IAA biosynthetic enzyme assay

Wild-type or mutated recombinant MBP-YUC6 proteins (10 μg) were incubated in a reaction buffer containing 10 mM NADPH, 2 mM IPA and 50 mM potassium phosphate (pH 6.5) for 4 h at 30 °C. Resulting IAA amounts were detected as a YUC activity using Ehrlich reagent (*p*-(dimethylamino)benzaldehyde; Sigma) at 585 nm on ultraviolet–visible spectrophotometer (Beckman Coulter)^65^,^66^.

### Insulin disulfide reductase assay

Insulin disulfide reductase activity was measured using 10 μM insulin (Sigma-Aldrich) with 1 mM DTT (Bioshop) as a reductant in reaction mixture containing 50 mM potassium phosphate (pH 8.0) and 2 mM EDTA. The reaction was initiated by the addition of varying amounts of recombinant YUC6 proteins (as MBP-fusion protein), and the activity was monitored for 30 min at 650 nm using an ultraviolet–visible spectrophotometer (Beckman Coulter) with slight modification^67^.

### Holdase chaperone assay

Purified Arabidopsis mitochondrial MDH (EC 1.1.1.37)^68^ and purified recombinant wild-type and mutant YUC6 proteins at the indicated molar ratios were incubated in 40 mM HEPES (pH 7.5) buffer at 45 °C. The absorbance at 340 nm, indicating aggregation of MDH, was monitored for 30 min using a Beckman DU-800 spectrophotometer (Beckman Coulter) attached to a thermostatic cell holder assembly^58^,^60^,^68^.

### Measurement of H_2_O_2_ content and activity of peroxidase and catalase

Two- and three-week-old plant tissues were harvested and frozen after treatment with MV (10 μM) and treatment with drought (air-drying) in the same manner as water-loss assay for 4 h, respectively. Frozen tissues were ground in liquid nitrogen. Ground tissues (50 mg) were soaked in 20 mM potassium phosphate buffer (pH 6.5) on ice. The extracts were clarified by centrifugation at 13,500*g* for 15 min at 4 °C. H_2_O_2_ concentration and peroxidase activity were measured in the supernatants using an Amplex Red hydrogen peroxide/peroxidase assay kit according to the manufacturer's instructions (Molecular Probes). Fluorescence was detected by a spectrofluorometer (Molecular Device). Catalase activity was measured with same plant extracts using the Amplex Red catalase assay kit according to the manufacturer's instructions (Molecular Probes).

### Histochemical staining for H_2_O_2_ and ROS

H_2_O_2_ accumulation was visualized with DAB. Detached leaves were floated on DAB solution (1 mg ml^−1^ in water, pH 3.8) for 4 h and then photographed.

ROS accumulation was visualized with H_2_DCF-DA. Seedlings (5-day-old) treated with or without MV as described under ‘DAB staining' were incubated for 30 min with 10 mM H_2_DCF-DA at 4 °C. Seedlings were then washed with 10 mM MES, 0.1 mM KCl and 0.1 mM CaCl_2_ (pH 6.0) and were incubated for 1 h at 22 °C. Fluorescence (excitation 488 nm and emission 522 nm) was detected using confocal microscopy (Olympus).

### Auxin quantification

Endogenous IAA was extracted in 0.5 ml of acetonitrile:formic acid:distilled water (16:1:3, v/v/v) from 3.5-week-old seedlings (0.20 g fresh-weight) with a Multi-Beads Shocker (model MB755U, Yasui Kikai Co., Osaka, Japan), after spiking 10 ng of ^13^C_6_-IAA (ICON Isotope Services Inc., Summit, NJ, USA) as an internal standard. The extraction of IAA was repeated independently three times with different plant materials. Its successive purification was performed with two cartridge columns for solid-phase extraction (OASIS MCX and MAX, 60 mg; Waters, Milford, MA, USA)^69^. The quantification was performed with a Waters Xevo TQ ESI mass spectrometer (Waters) linked with a Waters Acquity UPLC system carrying a BEH C18 column (1.7 μm, 2.1 × 50 mm, Waters). Multiple reaction-monitoring transitions (positive mode) were as follows: IAA, *m/z* 176.2>130.1 and 130.1>103.1; ^13^C_6_-IAA, *m/z* 182.2>136.2 and 136.2>109.2. The MassLynx 4.1 software (Waters) was used for data acquisition.

### GUS staining

Two-week-old soil-grown seedlings were incubated overnight in staining buffer (50 mM Tris-Cl pH 7.5, 0.2% Triton X-100, 1 mM 5-Bromo-4-chloro-3-indolyl β-D-glucuronic acid (X-gluc)) at 37 °C in the dark. Then, the seedlings were incubated overnight in 100% EtOH at room temperature to remove chlorophyll.

### RT–PCR and quantitative RT–PCR

For quantitative PCR, 2- or 3-week-old plants were harvested and ground in liquid nitrogen. Total RNA was extracted using TRIzol reagent (Qiagen). cDNA was synthesized using a oligo dT primer and reverse transcriptase (Solgent) at 42 °C for 60 min. Equal amounts of cDNA were used as templates for PCR amplification. Specific transcripts were amplified with gene-specific forward and reverse primers (Supplementary Table 2) using a step cycle programme on a Quantifast SYBR Green PCR Kit (Qiagen). Quantitative PCR measurements were performed on three biological repeats. Amplification curves and gene expression were normalized to the housekeeping gene *TUBULIN*, used as an internal standard.

## Additional information

**How to cite this article:** Cha, J.-Y. *et al*. A novel thiol-reductase activity of *Arabidopsis* YUC6 confers drought tolerance independently of auxin biosynthesis. *Nat. Commun.* 6:8041 doi: 10.1038/ncomms9041 (2015).

## Supplementary Material

Supplementary Information Supplementary Figures 1-7 and Supplementary Tables 1-2
