# Supplementary material for: A novel thiol-reductase activity of Arabidopsis YUC6 confers drought tolerance independently of auxin biosynthesis
Source: Nat Commun. 2015 Aug 28;6:8041. doi: 10.1038/ncomms9041 (PMC4560777; doi:10.1038/ncomms9041)
Supplement: Supplementary Information — Supplementary Figures 1-7 and Supplementary Tables 1-2 [file ncomms9041-s1.pdf]

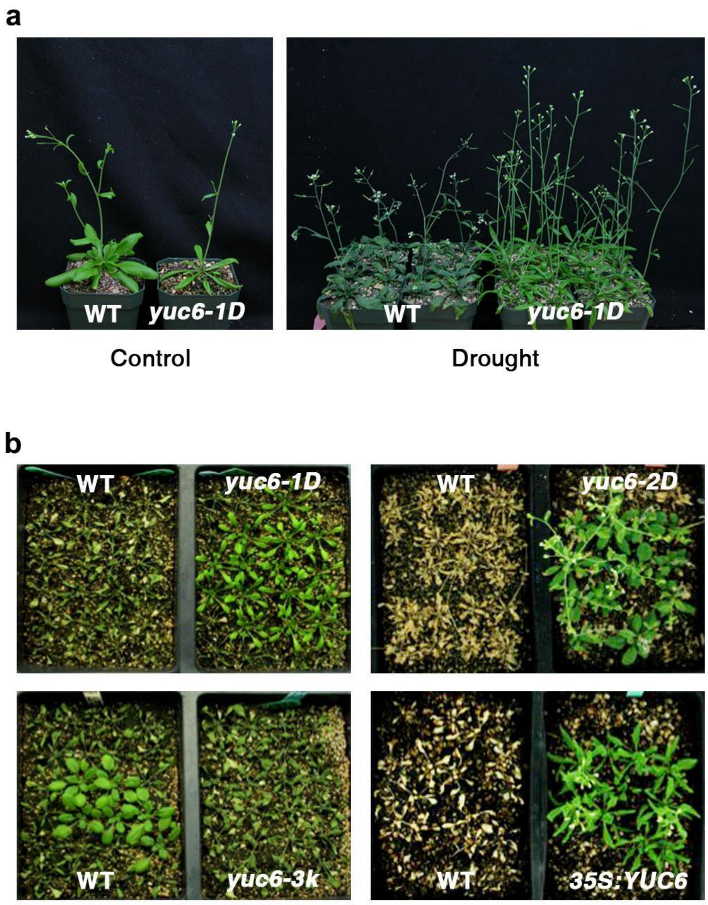

Supplementary Figure 1 | Overexpression of *YUC6* increases drought tolerance.

(**a** and **b**) Comparison of drought tolerance symptoms. (**a**) Shown are wild-type (WT, Col-*gl*) and *yuc6-1D* plants grown under well-watered conditions for 3 weeks (Control) and thereafter without watering for 14 days (Drought). (**b**) Shown are soil grown plants grown under well-watered conditions for 3 weeks and then allowed to grow without irrigation. Background ecotype of *yuc6-1D* and *yuc6-2D* is Col-*gl*, and that of *yuc6-3k* and *35S:YUC6* is Col-0. The *YUC6* overexpression (*yuc6-1D*, *yuc6-2D* and *35S::YUC6*) and their control WT plants were photographed after 12 days of drought. The loss-of function *yuc6-3k* mutant and its WT control were photographed after 10 days of drought. Isogenic WT lines were used for the comparisons. All plant materials have been described[5].

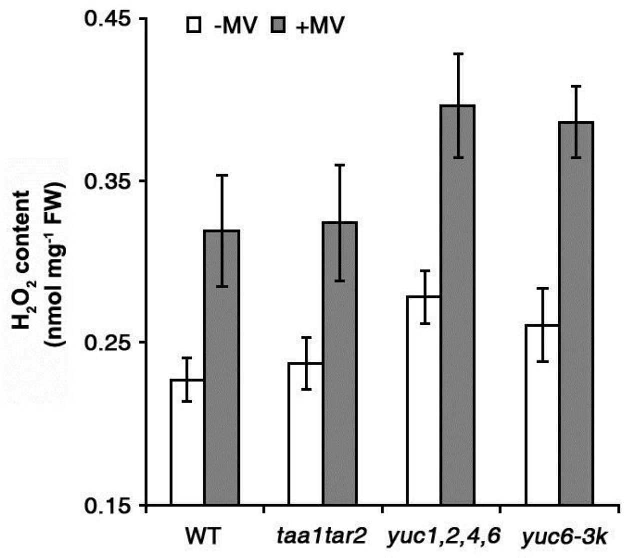

Supplementary Figure 2 | YUC proteins, but not auxin, are involved in ROS control under oxidative stress.

Three-week-old auxin deficient mutants, *taa1 tar2*, quadruple *yuc1,2,4,6*, and loss-of function *yuc6-3k* plants were untreated (-MV) or exposed to 10 µM methyl viologen (+MV) for 4 h. Quantification of H_2O_2 was done as described in Methods. Data represent means±SE, n=3.

Supplementary Figure 3

```
Q8VZ59|YUC6_ARATH   MDFCWKREMEGKLAHDH-----R--------------------------GMTSPRRICVV 29
Q39242|NTRA_ARATH   --MCWISMSQSRFIIKSLFSTAGGFLLGSALSNPPSLATAFSSSSSSSSAAAAVDMETHK 58
A9LN30|NTR2_HORVD   ---------------------------------------------------MEGSAAAPLR 10
P0A9P4|TRXB_ECOLI   ------------------------------------------------------MGTTKH 6
Q8KCB2|FENR_CHLTE   ----------------------------------------------MLDIHNPATDHHDM 14
                          FAD
Q8VZ59|YUC6_ARATH   TGPVIVGAGPSGLATAACLKERGITSVLLERSNCIASLWQLKTYDRLHLHLPKQFCELPI 89
Q39242|NTRA_ARATH   TKVCIVGSGPAAHTAAIYASRAELKPLLFEGW-------MAND-IAPGGQLTTT---TDV 107
A9LN30|NTR2_HORVD   TRVCIIGSGPAAHTAAIYAARAELKPVLFEGW-------MAND-IAAGGQLTTT---TDV 59
P0A9P4|TRXB_ECOLI   SKLLILGSGPAGYTAAVYAARANLQPVLITG-------------MEKGGQLTTT---TEV 50
Q8KCB2|FENR_CHLTE   RDLTIIGGGPTGIFAAFQCGMNNISCRIIESM------------PQLGGQLAALYPEKHI 62
                    *:*.**:.  :*      :  ::                 :*      :

Q8VZ59|YUC6_ARATH   IPFPGDFPTYPTK---QQFIEYLEDYARRFDIKPEFNQTVESA-AFDENLGMWRVTSVGE 145
Q39242|NTRA_ARATH   E----NFPGFPEGILGIDIVEKFRKQSERFGTTIFTET-VNKV-DFS--SKPFKL---FT 156
A9LN30|NTR2_HORVD   E----NFPGFPTGIMGIDLMDNCRAQSVRFGTNILSET-VTEV-DFS--ARPFRV---TS 108
P0A9P4|TRXB_ECOLI   E----NWPGDPNDLTGPLLMERMHEHATKFETEIIFDH-INKV-DLQ--NRPFRL---NG 99
Q8KCB2|FENR_CHLTE   Y----DVAGFPE-VPAIDLVESLWAQAERYNPDVVLNETVTKYTKLD--DGTFET---RT 112
                     :   *      :::    : ::     :  : .  :.     :.

Q8VZ59|YUC6_ARATH   EGTTEYVCRWLVAATGENAEPV--VPRF-EGMDKFAAAGVVKHTCHYKTGGDFAGKRVLV 202
Q39242|NTRA_ARATH   DSRT-VLADSVIISTGAVAKRL-SFTGSGEGNGGFWNRGISACAVCDGAAPIFRNKPLVV 214
A9LN30|NTR2_HORVD   DSTT-VLADTVVVATGAVARRL-YFSGS----DTYWNRGISACAVCDGAAPIFRNKPIAV 162
P0A9P4|TRXB_ECOLI   DNGE-YTCDALIIATGASARYL-GLPSE----EAFKGRGVSACATCDGF--FYRNQKVAV 151
Q8KCB2|FENR_CHLTE   NTGNVYRSRAVLIAAGLGAFEPRKLPQLGNI-DHL--TGSS-VYYAVKSVEDFKGKRVVI 168
                 :     . :: ::*  *    .           *           :  : : :
                      NADP
Q8VZ59|YUC6_ARATH   VGCGNSGMEVCLDLCNFGAQPSLVVRDAVHVLPREM----LGTSTFGLSMFLLKWLPIRL 258
Q39242|NTRA_ARATH   IGGGDSAMEEANFLTKYGSKVYIIHRRDTFRASKIMQQRALSNPKI-----EVIWNS-AV 268
A9LN30|NTR2_HORVD   IGGGDSAMEEGNFLTKYGSQVYIIHRRNTFRASKIMQARALSNPKI-----QVVWDS-EV 216
P0A9P4|TRXB_ECOLI   IGGGNTAVEEALYLSNIASEVHLIHRRDGFRAEKILIKRLMDKVENGNI--ILHTNR-TL 208
Q8KCB2|FENR_CHLTE   VGGGDSALDWTVGLIKNAASVTLVHRGHEFQGHGKTAHEVERARANGTI--DVYLET-EV 225
                 :* *::.::   * : .:.  :: *  .                   :     :

Q8VZ59|YUC6_ARATH   VDRFLLVVSRFILGDTTLLGLNRPRLGPLELKNISGKTPVLDVGTLAKIKTGDIKVCSGI 318
Q39242|NTRA_ARATH   ---------VEAYGDENG-----RVLGGLKVK-N-------------------------- 287
A9LN30|NTR2_HORVD   ---------VEAYGGAGG-----GPLAGVKVK-N-------------------------- 235
P0A9P4|TRXB_ECOLI   ---------EEVTGDQMG-------VTGVRLRDT-------------------------- 226
Q8KCB2|FENR_CHLTE   ---------ASI--EESN-----GVLTRVHLRSS-------------------------- 243
                                      : :.::

Q8VZ59|YUC6_ARATH   RRLKRHEVEFDNGKTERFDAIILATGYKSNVPSWLKENKMFSKKDGFPIQEF----PEGW 374
Q39242|NTRA_ARATH   -------VVTGDVSDLKVSGLFFAIGHEPATKF-LDGQ-LELDEDGYVVTKP----GTTK 334
A9LN30|NTR2_HORVD   -------LVTGEVSDLQVSGLFFAIGHEPATKF-LNGQ-LELHADGYVATKP----GSTH 282
P0A9P4|TRXB_ECOLI   -------QNSDNIESLDVAGLFVAIGHSPNTAI-FEGQ-LELEN-GYIKVQSGIHGNATQ 276
Q8KCB2|FENR_CHLTE   ---------DGSKWTVEADRLLILIGFKSNLGPLARWD-LELYENALVV------DSHMK 287
                          .       ::. *..        : :    .

Q8VZ59|YUC6_ARATH   RGECGLYAV----------GFTKRGISGASMDAKRIAEDIHKCWKQDEQVKKI------- 417
Q39242|NTRA_ARATH   TSVVGVFAAGDVQDKKYRQAITAAGT--GCMAALDAEHYL----QEIGS----------Q 378
A9LN30|NTR2_HORVD   TSVEGVFAAGDVQDKKYRQAITAAGS--GCMAALDAEHYL----QEVGA----------Q 326
P0A9P4|TRXB_ECOLI   TSIPGVFAAGDVMDHIYRQAITSAGT--GCMAALDAERYL----DGLAD----------A 320
Q8KCB2|FENR_CHLTE   TSVDGLYAAGDIAYYPGKLKIIQTGLSEATMAVRHSLSYI----KPGEKIRNVFSSVKMA 343
                  . *::*.         :   *  . * .     :    .

Q8VZ59|YUC6_ARATH   -----------------
Q39242|NTRA_ARATH   EGKSD------------ 383
A9LN30|NTR2_HORVD   VGKSD------------ 331
P0A9P4|TRXB_ECOLI   K---------------- 321
Q8KCB2|FENR_CHLTE   KEKKAAEAGNATENKAE 360
```

Supplementary Figure 3 | Conservation of FAD and NADP binding sites are evolutionarily conserved in thiol-reductases and ferrodoxin-reductases. Sequences of thioredoxin-reductases from *A. thaliana* (NTRA), barley (NTR2) and *E. coli* (TRXB) were aligned with YUC6. In addition, the ferredoxin-NADP reductase of the green alga *Chlorobium tepidum* (FENR) was aligned using the Clustal-O program tool at the UniProt web site. UniProt protein codes are given for each peptide. Essential Gly residues in FAD and NADP binding sites are marked in *yellow*.

Cys residues at the active sites (CxxC) of Arabidopsis NTRA, barley NTR2 and *E. coli* TrxB thioredoxin reductases are marked in *green*. Cys-63 in the conserved motif CIASLW, and Cys-85 in the conserved motif C(E/Q)LP of YUC6 are marked in *blue*.

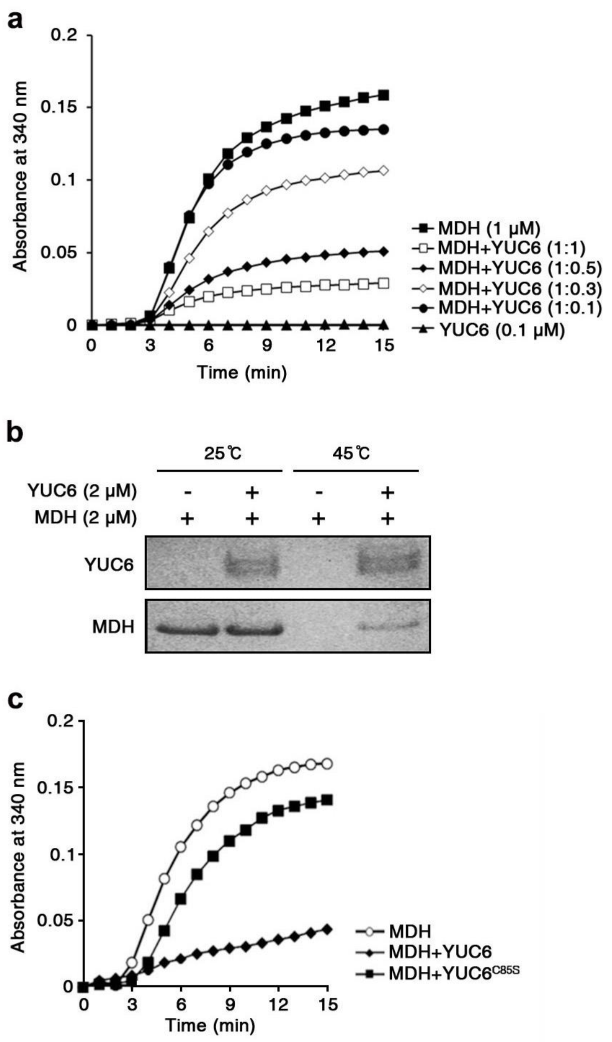

Supplementary Figure 4 | YUC6 exhibits holdase chaperone activity in a Cys-85 dependently.

Holdase chaperone activity of YUC6 was determined spectrophotometically (**a** and **c**) and by SDS-PAGE migration (**b**). (**a**) A solution containing purified Arabidopsis malate dehydrogenase (MDH, 1 μM) and purified recombinant MBP-tagged YUC6 protein in the indicated molar ratios was heated at 45 ^{o}C for the indicated time period. Thermal aggregation of MDH was monitored as increase in Abs_{340nm}. (**b**) MDH (2 μM) was incubated in the absence or presence of YUC6 (2 μM) for 15 min at 25 or 45 ^{o}C. After centrifugation, soluble fractions were rescued and separated on SDS-PAGE. (**c**) Holdase chaperone activity was measured as in Supplementary Figure 4a using a 1:1 molar ratio of MDH and indicated recombinant MBP-tagged YUC6 and $YUC6^{C85S}$ proteins.

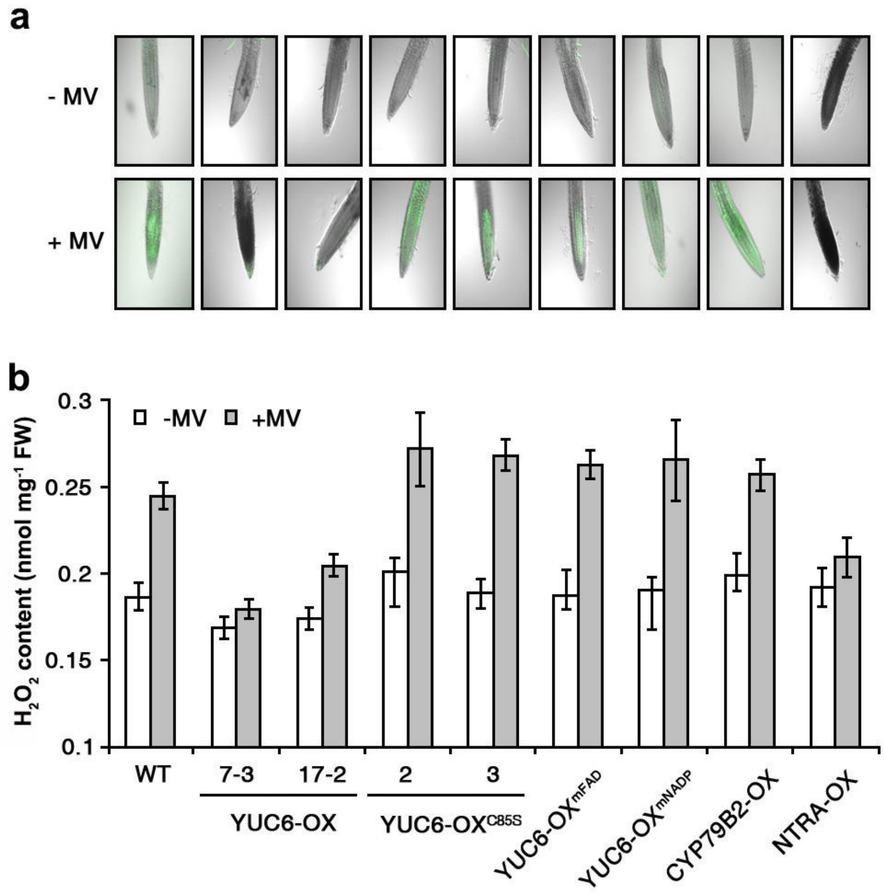

Supplementary Figure 5 | Cys-85 is essential for reduced ROS accumulation under oxidative stress in *YUC6* overexpression plants.

(**a**) Shown are roots of 5-day-old plants grown on MS agar, treated without (-MV) or with 10 µM MV (+MV) for 4 h, and stained with H_2DCF-DA for H_2O_2 detection. (**b**) Three-week-old aerial parts of plants were treated with MV as in (**a**) and H_2O_2 contents were quantitatively analyzed. Data represent means±SE, n=3.

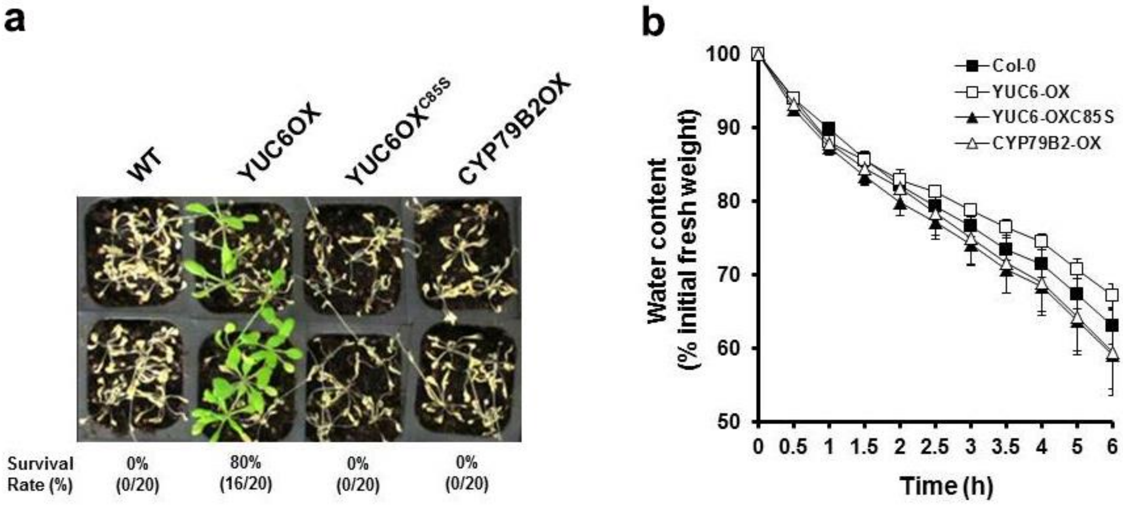

Supplementary Figure 6 | Auxin-overproduction driven by the overexpression of *CYP79B2* is not involved for the drought tolerance.

Col-0 plants (WT) were compared to YUC6-OX, YUC6-OX^{C85S}, and CYP79B2-OX transgenics in the same background. (**a**) Drought survival assay. Three-week-old well-watered plants were left for 7 days without irrigation. At 4 days after resuming irrigation, the plants were photographed, and the survival rate (percent) in each sample was quantified and is given at the bottom of the picture. (**b**) Water loss assay. The assay was performed as described in Figs. 1b and 5b.

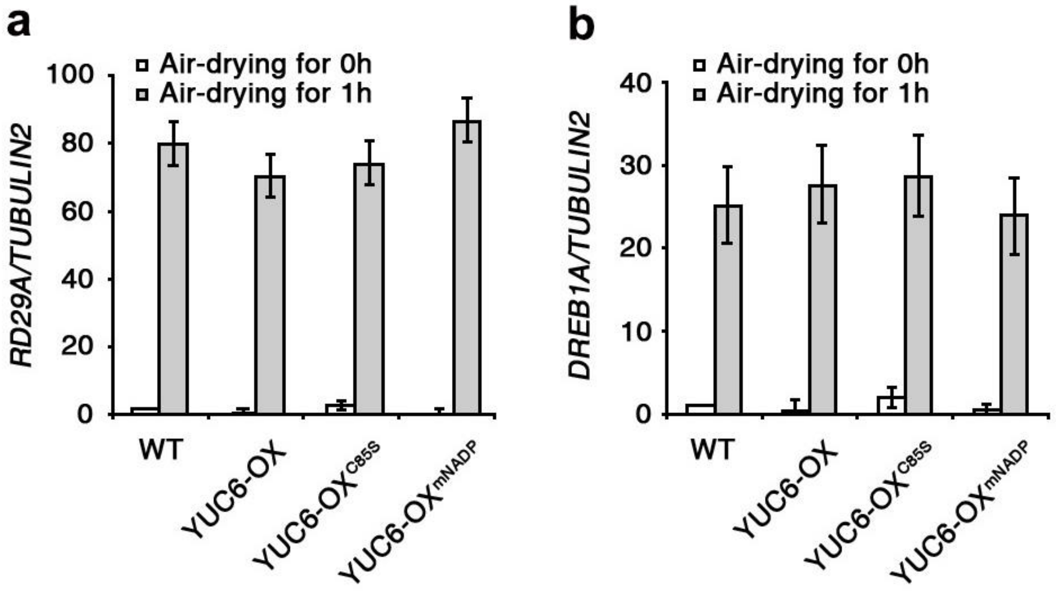

Supplementary Figure 7 | Expression of the drought-responsive genes *RD29A* and *DREB1A* is not affected by overexpression of *YUC6*.

RD29A (**a**) and *DREB1A* (**b**) transcript levels were measured by qRT-PCR in total RNA from aerial parts of 3-week-old soil grown WT (Col-0), YUC6-OX, YUC6-OX^{C85S} and YUC6-OX^{mNADP} plants that were allowed to air-dry naturally for the indicated time periods. The expression levels were normalized compared to those of *TUB*. Bars represent means±SE, n=3.

Supplementary Table 1 Expression of ROS homeostasis genes is elevated in *yuc6-1D*.

AGI ID	Protein and GO Function	Mean log[2]	Fold
At5g38000	Zinc-binding dehydrogenase, Oxidoreductase	1.181	2.27
At5g37940	Zinc-binding dehydrogenase, Oxidoreductase	1.138	2.20
At5g17000	Zinc-binding dehydrogenase, Oxidoreductase	0.568	1.48
At4g36090	2-oxoglutarate and Fe(II)-dependent oxygenase, Oxidoreductase	0.295	1.23
At5g59540	2-oxoglutarate and Fe(II)-dependent oxygenase, Oxidoreductase	0.757	1.69
At3g46500	2-oxoglutarate and Fe(II)-dependent oxygenase, Oxidoreductase	0.844	1.80
At3g46490	2-oxoglutarate and Fe(II)-dependent oxygenase, Oxidoreductase	0.890	1.85
At3g28480	2-oxoglutarate and Fe(II)-dependent oxygenase, Oxidoreductase	0.269	1.20
At2g18150	Peroxidase, Oxidation reduction, heme and metal binding	0.423	1.34
At4g32580	Thioredoxin, Redox homeostasis	0.259	1.20

Microarray hybridization was carried in a previous study[5]. Fold induction of ROS homeostasis genes in *yuc6-1D* versus the wild-type are listed.

Supplementary Table 2 Primer sequences used in this study.

Name	Primer sequence (5' to 3')	Purpose
YUC6	F: AAAAAGCAGGCTTCATGGATTTCTGTTGGAAGAG<br>R: AGAAAGCTGGGTCGATTTTTTTTACTTGCTCGTC	Gateway cloning
YUC6^{C85S}	F: CTTCCTAAACAATTCTCTGAACTTCCGATTATA<br>R: TATAATCGGAAGTTCAGAGAATTGTTTAGGAAG	Mutagenesis
YUC6^{mFAD}	F: CCGGTGATCGTAGGCGCCGGACCGTCGGTACTAG<br>R: CATGCTGCCGTGGCTAGTACCGACGGTCCG	Mutagenesis
NTRA	F: GCGGATCCATGAGCCAGTCAAGATTC<br>R: GCCTCGAGTCAATCACTCTTACCCTC	Cloning
IAA1	F: ATGGAAGTCACCAATGGGGCTTAACCT<br>R: TCATAAGGCAGTAGGAGCTTCGGATC	qRT-PCR
RD29A	F: ATCACTTGGCTCCACTGTTGTTC<br>R: ACAAAACACACATAAACATCCAAAGT	qRT-PCR
DREB1A	F: TGCGTTGGCGTTTCAGGATG<br>R: CAAACTCGGCATCTCAAACATCG	qRT-PCR
TUB	F:AGCAAATGTGGGACTCCAAG<br>R:CACCTTCTTCATCCGCAGTT	qRT-PCR
CYP79B2	F: AAAAAGCAGGCTTCATGAACACTTTTACCTCAAA<br>R: AGAAAGCTGGGTCCTTCACCGTCGGGTAGAG	Gateway cloning
